# RNA-seq data exploration after trypanosome RNA-binding protein UBP1 expression is altered by CRISPR-Cas9 gene editing and overexpression

**DOI:** 10.1016/j.dib.2024.110156

**Published:** 2024-02-06

**Authors:** Karina B. Sabalette, Vanina A. Campo, José R. Sotelo-Silveira, Pablo Smircich, Javier G. De Gaudenzi

**Affiliations:** a Instituto de Investigaciones Biotecnológicas, Universidad Nacional de San Martín - Consejo Nacional de Investigaciones Científicas y Técnicas, 1650 General San Martín, Prov. de Buenos Aires, Argentina; b Escuela de Bio y Nanotecnologías (EByN), Universidad Nacional de San Martín, 1650 General San Martín, Prov. de Buenos Aires, Argentina; c Instituto de Investigaciones Biológicas Clemente Estable, Av. Italia 3318, Montevideo, CP 11600, Uruguay; d Instituto de Biología, School of Sciences, Universidad de la República, Montevideo, Uruguay

**Keywords:** RNA-binding protein, Transcriptomics, Trypanosoma cruzi, Differential gene expression, Parasite differentiation

## Abstract

Previous studies have shown that overexpression of the *Trypanosoma cruzi* U-rich RNA-binding protein 1 (TcUBP1) in insect-dwelling epimastigotes results in a gene expression pattern resembling that of the infective form of the pathogen. Here, we used CRISPR-Cas9-induced edition of TcUBP1 and full-length protein overexpression in epimastigote cells to monitor transcriptomic changes during the epimastigote-to-metacyclic trypomastigote stage transition of *T. cruzi*. This dataset includes the bioinformatics analysis of three different RNA-seq samples, each with three biological replicates, showing differential mRNA abundances. The current transcriptome report has the potential to shed light on the quantitative variances in the expression of significant

up- or down-regulated mRNAs as a consequence of the levels of the UBP1 protein. Raw data files were deposited at the NCBI Sequence Read Archive - SRA at http://ncbi.nlm.nih.gov/Traces/sra/sra.cgi with accession numbers PRJNA907231 and PRJNA949967.

Specifications Table

**Table:** 

Subject	Transcriptomics Molecular Parasitology
Specific subject area	Transcriptome dataset of TcUBP1-CRISPR/Cas9 gene editing and TcUBP1-GFP overeexpressing epimastigote cells in *Trypanosoma cruzi*
Data format	Raw, Analyzed
Type of data	Table, Figure
Data collection	High-throuput sequencing carried out by BGI using using 2 × 100 PE chemistry on DNBSeq platform
Data source location	Buenos Aires, Argentina
Data accessibility	RNA-seq data from this study has been submitted to the NCBI Sequence Archive - SRA at http://ncbi.nlm.nih.gov/Traces/sra/sra.cgi with accession numbers PRJNA907231 and PRJNA949967 (available at https://www.ncbi.nlm.nih.gov/bioproject/PRJNA907231 and https://www.ncbi.nlm.nih.gov/bioproject/PRJNA949967).

## 1 Value of the Data

• This is the first transcriptome study of *Trypanosoma cruzi* RNA-binding protein UBP1-overexpressing and knockdown parasites.
• The RNA-Seq dataset underlines the quantitative differences of significant up- or down-regulated mRNAs as a direct effect of UBP1 protein levels.
• Further gene expression analysis will provide a data source for understanding of how *T. cruzi* epimastigotes differentiate to infective metacyclic trypomastigotes.

## 2 Background

The present study focused on the U-rich RNA-binding protein 1 (UBP1, TcCLB.507093.220), one of the first trypanosome RNA-recognition motif-containing proteins to be described. Remarkably, *UBP1* gene is encoded in a single stable dicistronic unit and its protein product regulates the abundance of several genes that have U-rich elements in *Trypanosoma cruzi* [1,2]. We have already shown that when UBP1 is overexpressed in insect-dwelling epimastigotes, the microorganism shows an expression pattern that looks like the infective form [3]. To further investigate the regulatory role of this protein, we used the CRISPR-Cas9 technology to generate a population of parasites that did not express this protein. However, we obtained an N-terminal mutated protein that is significantly less expressed than the endogenous form found in normal cells [4].

## 3 Data Description

**Fig. 1:**
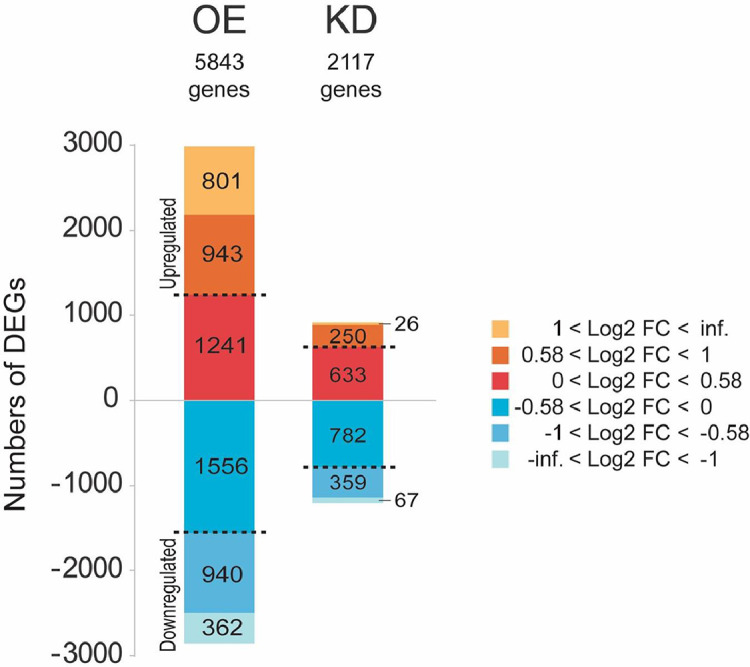
A bar graph for the number of genes identified in the two conditions. Stacked bar charts show the distribution of the number of significant genes and DEGs identified in each pairwise comparison between OE (UBP1-OE) and KD (UBP1mut-KD) *versus* WT.

This data describes a transcriptomic experiment to investigate the differential gene expression profiles between the *T. cruzi* N-terminal mutated UBP1 knockdown [4] and full-length protein overexpression in epimastigote cells. The experimental design consists of three high-throughput sequencing samples with three biological replicates each of wild-type RNA-binding protein TcUBP1 (WT), UBP1-GFP tetracycline-induced epimastigotes for four days (UBP1-OE), and CRISPR/Cas edited epimastigotes (UBP1mut-KD). Differential gene analysis was conducted using DESeq2 [5], with the criteria of at least 2-fold change (|Log2 fold change| > 1, FDR-adjusted *p* value < 0.05). The expression values listed in Supplementary Table 1 show the average Log2 fold change for all mapped genes in UBP1-OE or UBP1mut-KD *versus* WT. TcUBP1 overexpression in epimastigote cells led to a significant increase in the abundance of 801 genes and a downregulation of 362 genes. In the population of UBP1mut-KD parasites, a significant decrease of 26 genes and an increase of 67 genes was observed (Fig. 1). Next, the transcriptome dataset was examined to check the amount of UBP1 transcript levels within the two conditions, with UBP1 being the experimental internal control of our RNA-Seq analysis. As expected, the Log2 fold change obtained for UBP1-OE/WT was 6.16 (FDR-adjusted *p* value = 2.1E-84) and the Log2 fold change of UBP1mut-KD/WT was -0.90 (FDR-adjusted *p* value = 2.1E-02). In addition, the oppositely affected genes after UBP1 overexpression and knockdown were recently reported by Sabalette *et al.* (see Ref. [4]). Raw data is available through NCBI's Sequence Read Archive at http://www.ncbi.nlm.nih.gov/sra.cgi with accession numbers: PRJNA907231 and PRJNA949967.

## 4 Experimental Design, Materials and Methods

Control wild-type UBP1, four-day tetracycline-induced UBP1 parasites [6], and UBP1-CRISPR/Cas9 edited cells [4] were cultured in epimastigote form conditions at 28 ºC. Total RNA from three biological replicates was prepared from approximately 10^7^ epimastigote cells of each sample (WT, UBP1-OE, and UBP1mut-KD) using the TRIzol reagent following the manufacturer's instructions (Invitrogen). Isolation of poly(A)+ mRNA, library preparation, and deep-sequencing on an DNBSeq platform were performed at BGI. 100 nt reads were mapped to the *T. cruzi* reference genome CL Brener Esmeraldo-like strain (TriTrypDB-59_TcruziCLBrenerEsmeraldo-like_Genome.fasta), as described [3]. Differential gene analysis was conducted using DESeq2 [5]. All RNA-seq raw data files for WT, UBP1-overexpression, and UBP1-CRISPR/Cas9 edited samples, used in this study, are available as FASTQ files of 100 bp paired-end reads in the National Center for Biotechnology Information (NCBI) Sequence Read Archive (SRA) database with the following study numbers: PRJNA907231 and PRJNA949967.

## Limitations

Not applicable.

## Ethics statement

The authors have read and follow the ethical requirements for publication in Data in Brief and confirming that the current work does not involve human subjects, animal experiments, or any data collected from social media platforms.

## Declaration of generative AI and AI-assisted technologies in the writing process’

During the preparation of this work the authors used ChatGPT (OpenAI) in order to improve language and readability. After using this tool/service, the authors reviewed and edited the content as needed and take full responsibility for the content of the publication.

## CRediT authorship contribution statement

**Karina B. Sabalette:** Methodology. **Vanina A. Campo:** Methodology, Formal analysis. **José R. Sotelo-Silveira:** Data curation, Formal analysis. **Pablo Smircich:** Data curation, Formal analysis, Funding acquisition. **Javier G. De Gaudenzi:** Formal analysis, Visualization, Writing – original draft, Conceptualization, Funding acquisition, Supervision.

## Data Availability

RNA-seq of TcUBP1 knockdown in Trypanosoma cruzi CL-Brener epimastigotes (Original data) (SRA) RNA-seq of TcUBP1 transgenic Trypanosoma cruzi CL-Brener epimastigotes (Original data) (SRA)

## Appendix Supplementary materials

Table S1. RNA-seq expression Log2 values for UBP1-OE and UBP1mut-KD *versus* WT control sample. Image, application 1
